# A Novel Embryo Phenotype Associated With Interspecific Hybrid Weakness in Rice Is Controlled by the MADS-Domain Transcription Factor *OsMADS8*

**DOI:** 10.3389/fpls.2021.778008

**Published:** 2022-01-05

**Authors:** Sun Ha Kim, Shi-Dong Ji, Hyun-Sook Lee, Yun-A Jeon, Kyu-Chan Shim, Cheryl Adeva, Ngoc Ha Luong, Pingrong Yuan, Hyun-Jung Kim, Thomas H. Tai, Sang-Nag Ahn

**Affiliations:** ^1^Department of Agronomy, College of Agriculture & Life Sciences, Chungnam National University, Daejeon, South Korea; ^2^LG Chem., Ltd., Seoul, South Korea; ^3^Crops Pathology and Genetics Research Unit, USDA-ARS, Davis, CA, United States; ^4^Department of Plant Sciences, University of California, Davis, Davis, CA, United States

**Keywords:** hybrid weakness, dark tip embryo, rice, floral organ determination, seed germination, abscisic acid

## Abstract

A novel hybrid weakness gene, *DTE9*, associated with a dark tip embryo (DTE) trait, was observed in CR6078, an introgression line derived from a cross between the *Oryza sativa* spp. *japonica* “Hwayeong” (HY) and the wild relative *Oryza rufipogon*. CR6078 seeds exhibit protruding embryos and flowers have altered inner floral organs. *DTE9* was also associated with several hybrid weakness symptoms including decreased grain weight. Map-based cloning and transgenic approaches revealed that *DTE9* is an allele of *OsMADS8*, a MADS-domain transcription factor. Genetic analysis indicated that two recessive complementary genes were responsible for the expression of the DTE trait. No sequence differences were observed between the two parental lines in the *OsMADS8* coding region; however, numerous single nucleotide polymorphisms were detected in the promoter and intronic regions. We generated overexpression (OX) and RNA interference (RNAi) transgenic lines of *OsMADS8* in HY and CR6078, respectively. The *OsMADS8-*OX lines showed the dark tip embryo phenotype, whereas *OsMADS8*-RNAi recovered the normal embryo phenotype. Changes in gene expression, including of ABCDE floral homeotic genes, were observed in the *OsMADS8*-OX and *OsMADS8*-RNAi lines. Overexpression of *OsMADS8* led to decreased expression of *OsEMF2b* and ABA signaling-related genes including *OsVP1/ABI3*. HY seeds showed higher ABA content than CR6078 seeds, consistent with *OsMADS8/DTE9* regulating the expression of genes related ABA catabolism in CR6078. Our results suggest that *OsMADS8* is critical for floral organ determination and seed germination and that these effects are the result of regulation of the expression of *OsEMF2b* and its role in ABA signaling and catabolism.

## Introduction

Hybrid weakness (HW) or hybrid breakdown is a postembryonic barrier in the F_2_ or later generations of interspecific or intraspecific crosses (Stebbins, 1950). In rice, F_1_ hybrids between *indica* and *japonica* display various reproductive barriers including hybrid sterility (Oka, 1974; Ikehashi and Araki, 1986) and hybrid weakness (Oka, 1957; Sato and Morishima, 1987). Hybrid weakness, a form of hybrid incompatibility during the postembryonic stage of plant development, is frequently observed in many other plant species including *Arabidopsis thaliana* (Bomblies et al., 2007). Hybrid weakness manifests in rice through characteristic dwarfing, chlorotic phenotype, stunted growth, and poor seed setting (Ichitani et al., 2011; Chen et al., 2013). Nearly all studies have reported that hybrid weakness is controlled by the complementary interaction of unlinked loci (Fukuoka et al., 1998, 2005; Kubo and Yoshimura, 2002; Matsubara et al., 2007; Yamamoto et al., 2007). Recently, three causal genes for hybrid weakness at two loci, *Hwi1* and *Hwi2*, have been cloned in rice (Chen et al., 2014). *Hwi1*, a locus with two leucine-rich repeat receptor-like kinase (LRR-RLK) genes, induces hybrid weakness while *Hwi2* encodes a secreted putative subtilisin-like protease. When combined, *Hwi1* and *Hwi2* activate autoimmune responses in the basal nodes of hybrids, interrupting root formation and impairing shoot growth. Although hybrid weakness is associated with abnormal growth and development, there have been no reports indicating a role of plant hormones in regulating this disorder in rice.

In angiosperms, the flowers are typically comprised of sepals, petals, stamens, and pistils arranged in concentric whorls. The concept of whorls underlies the classical ABC model of floral organ development and, subsequently, the ABCDE model (Coen and Meyerowitz, 1991; Gutierrez-Cortines and Davies, 2000; Jeon et al., 2000; Honma and Goto, 2001; Lee et al., 2003; Yoshida and Nagato, 2011; Murai, 2013; Callens et al., 2018; Ali et al., 2019; Supplementary Figure S1). According to the ABC model, class A, B, and C genes together specify the identity of sepals, petals, stamens, and carpels (Angenent and Colombo, 1996; Pelaz et al., 2000). Since the early 2000s, many studies have reported that two additional classes of floral homeotic genes, D and E (SEPALLATA, *SEP*), are also necessary for specifying floral organ identity (Pelaz et al., 2000; Murai, 2013; Song et al., 2018). While the class D genes are crucial for ovule development, class E genes are required for specifying all floral organs. Other classes of genes involved in flower development include FRIZZY PANICLE (*FZP*), SUPERNUMERARY BRACT (*SNB*), MULTI-FLORET SPIKELET1 (*MFS1*), and APETALA2/ethylene-responsive factor (*AP2*/*ERF*) gene families (Komatsu et al., 2003; Lee et al., 2007a; Bai et al., 2016; Pasriga et al., 2019).

Most of the genes in the ABCDE model encode the MIKC_C_-type MADS-domain transcription factors (Riechmann and Meyerowitz, 1997; Egea-Cortines et al., 1999; Becker et al., 2000; Honma and Goto, 2001; Theissen, 2001). In rice, there are five different SEP-like genes (Arora et al., 2007): *OsMADS1* (Jeon et al., 2000; Chen et al., 2006), *OsMADS5* and *OsMADS34* (also called *OsMADS19*) are likely members of the SEP1/2/4 clade of genes, and *OsMADS7* and *OsMADS8* are SEP3-like genes (Kang and An, 1997; Zahn et al., 2005). The functions of the monocot SEP-like genes are unknown, except for *OsMADS1* (Agrawal et al., 2005; Prasad et al., 2005).

Several genes that are not classified as a family of *MADS* genes are related to determining floral organ identity. DROOPING LEAF (*DL*), a member of the YABBY gene family, controls the lemma development and carpel specification in rice (Nagasawa et al., 2003; Ishikawa et al., 2009; Li et al., 2011). OPEN BEAK (*OPB*) and DEGENERATED HULL1 (*DH1*) are required for the normal lemma and palea development (Horigome et al., 2009; Xiao et al., 2009). In *Arabidopsis*, the polycomb repressive complex 2 (*PRC2*) is known to regulate flower development through the inhibition of expression of the C function flower homologous gene, AGAMOUS (*AG*; Goodrich et al., 1997). The loss of either of the *PRC2* components or EMBRYONIC FLOWER 2 (*EMF2*) results in a mild floral phenotype in *Arabidopsis* (Deng et al., 2017). Although the function of plant *PRC2* is well-characterized in *Arabidopsis*, its specific function in other plant species remains unknown. Conrad et al. (2014) confirmed through transcriptome analysis that the E-function genes *OsMADS1*, *OsMADS6*, and *OsMADS34* were differentially expressed in wild-type and *emf2b* mutants.

It is well known that abscisic acid (ABA) promotes seed dormancy and suppresses seed germination and seedling growth. Chen et al. (2017) reported that *OsEMF2b* regulates seed dormancy and seedling growth by activating or inhibiting the expression of ABA signaling response genes. Downregulation of *OsEMF2b* expression leads to vivipary and a decrease in the expression level of *OsVP1*, an *ABI3* ortholog in rice. *ABI3*, a component of the ABA signal transduction pathway, plays a major role in seed development and dormancy inception. Disruption of the *OsVP1* transcription reduces the seed dormancy or viability (Chen et al., 2017).

In this study, a novel interspecific hybrid weakness gene, dark tip embryo (*DTE9*), was discovered in CR6078, an introgression line derived from a cross between the *Oryza sativa* spp. *japonica “*Hwayeong” (HY) and the wild relative *Oryza rufipogon*. In addition to presenting the typical symptoms of hybrid weakness in rice, such as dwarfing, plants exhibiting the DTE trait showed a significant association with several deleterious traits including the ripening ratio (i.e., fertility). We characterized the phenotypic and physiological properties of DTE as a newly discovered hybrid weakness. The genetic locus of DTE was analyzed, and the gene responsible for this phenotype was fine mapped within a 27.7 kb region on chromosome nine containing two candidate genes. Gene expression and transgenic analysis indicated that *OsMADS8*, a MADS-domain transcription factor, controls the DTE phenotype by affecting the expression of class A, B, C, D, and E floral homeotic genes as well as viviparous genes related to ABA biosynthesis or signaling.

## Materials and Methods

### Plant Materials

Previously, an introgression line population was developed from a cross between “Hwayeong” (*O. sativa* ssp. *japonica*), a Korean elite rice cultivar, and W1944 (*O. rufipogon* Griff.), a wild relative of cultivated rice from China (Lee et al., 2005). A DTE phenotype was observed in a line, CR6078 (BC_3_F_3_), which contained eight W1944 chromosome segments in the Hwayeong (HY) genetic background (Supplementary Figures S2, S3). CR6078 was backcrossed to HY, and the BC_1_ plants were self-pollinated to produce 204 BC_4_F_2:3_ lines. Preliminary mapping with eight simple sequence repeat (SSR) markers, one per W1944 segment, indicated that the gene for the DTE trait was located on chromosome 9 (designated *DTE9*) between RM553 and RM24637. Six BC_4_F_3_ plants that were heterozygous for the target region at RM553 and RM24637 were self-pollinated to produce a BC_4_F_4_ population for fine mapping *DTE9*. In total, 4,500 BC_4_F_4_ plants were genotyped with RM553 and RM24637, and 50 recombinants between the two markers were detected. These 50 BC_4_F_4:5_ recombinant lines were used to narrow down the location of the gene for *DTE9* by substitution mapping.

### Phenotype Evaluation

A total of 204 BC_4_F_3_, 4,500 BC_4_F_4_ plants, 50 BC_4_F_4_ recombinant lines, and the parents (HY and CR6078) were grown at the experimental field of Chungnam National University, Daejeon during the summers of 2008, 2009, and 2010, respectively. The 204 BC_4_F_3_ and 4,500 BC_4_F_4_ plants were planted at 15 cm intervals within and 30 cm intervals between the rows. The 50 BC_4_F_4_ lines were represented by a single row of 30-day-old seedlings planted at an interval of 15 cm within and 30 cm between the rows. The BC_4_F_3_ population was evaluated for ripening ratio (RR), 1,000-grain weight (TGW), and grain weight per plant (GW). RR, a measure of fertility, was calculated as a percentage of the number of filled spikelets divided by the number of spikelets per panicle. The number of spikelets per panicle was measured by averaging the values of three major panicles per plant. The grains were dried naturally after harvesting, and the partially filled or unfilled seeds were removed with water (i.e., density separation). The filled seeds were re-dried in an oven at 30°C for 24 h. The TGW was evaluated by measuring the weight of 100 randomly selected filled grains averaged over two replications. The grain yield per plant (GW) was evaluated by averaging the grain yield (g) at 13% moisture content of five plants.

The germination rate, measured as a percentage, was compared among the seeds of HY, W1944, CR6078, and *OsMADS8* transgenic plants. Germination tests were conducted as described by Nguyen et al. (2012) and Shim et al. (2019) with some modifications. Seeds were collected at 45 days after flowering. After breaking the seed dormancy at 50°C for 5 days, two sets of 30 seeds per line were germinated at 30°C under dark condition. Germination tests were replicated three times. Three panicles of HY and CR6078 were sampled at 1 or 2 days before flowering to determine pollen viability. Pollen collection and staining were carried out as described by Jiang et al. (2018). The anthers were crushed into powder and used to observe fertility with a light microscope. Pollen grains that were round and stained black were considered to be viable.

### DNA Extraction, Marker Development, and Quantitative Trait Loci Analysis

DNA extraction from the leaf tissues, PCR amplification of SSR markers, size separation using polyacrylamide gel electrophoresis, and marker detection by silver staining were performed as described by Shim et al. (2019). Silver staining kits were purchased from Bioneer Co., Daejeon, Korea.^1^ The linkage and orientation of the SSR markers were based on SSR maps (McCouch et al., 2002). Using the publicly available sequences for Nipponbare^2^ and 93-11^3^ reference genomes, insertion and deletion markers were developed using the PRIMER 3.0 program (Supplementary Table S1). The quantitative trait loci (QTL) were fine mapped by comparing the phenotypic means of the genotypic classes of recombinants within the target region using the analysis of variance (ANOVA) feature in the Data Desk 4.0 and based on the interval analysis (*p* ≤ 0.01 and/or LOD ≥ 3.0) for the markers within the target region using QGENE (Nelson, 1997).

### RNA Isolation and Quantitative Real-Time PCR

RNA was isolated from the seeds of the three lines (HY, W1944, and CR6078) and 7-day-old seedlings of transgenic plants using a NucleoSpin RNA kit (Macherey Nagel, Duren, Germany) according to the manufacturer’s instructions. Following reverse transcription into the first-strand cDNA with SmartGene Mixed cDNA synthesis kit (SJ Bioscience, Daejeon, Korea), real-time PCR was performed using a CFX Connect Real-Time System (Bio-Rad, Hercules, CA, United States). The real-time PCR protocol was as follows: 15 min at 95°C denaturation and enzyme activation, followed by 40 cycles at 95°C for 20 s, 60–55°C for 40 s (depending on the primer annealing temperature), and 72°C for 30 s. The rice tumor protein homolog (LOC_Os11g43900, *OsTMP*) was used for normalization, and the relative expression levels were compared using Student’s *t*-test and Tukey’s test. The primers used in this study are listed in Supplementary Table S1.

### Preparation of Constructs and Plant Transformation

To clone the full-length cDNA of *OsMADS8* gene from W1944, a pair of gene-specific primer (Forward: 5'-ATGGGGAGAGGGAGGGTGG-3' and Reverse: 5'-GGGGTAG CCATGTCGGCATG-3'), was synthesized based on the sequence of Nipponbare *OsMADS8* gene from the RAP-DB.^4^ The PCR product was cloned into the pGEM-T Easy vector (Promega, Madison, WI, United States) and was sequenced to confirm fidelity. To construct the plant expression vectors, we used the Gateway cloning system (Invitrogen, Carlsbad, CA, United States). The PCR products were used for the second PCR reaction with primers containing attB sites (5'-GTACAAAAAAGCAGGCTATGGGGAGAGGGAGGGTGG-3') and (5'-ACCACTTTGTACAAGAAAGC TGGGTCGGGGGTAGCCATGTCGGCATG-3'). The linear fragments flanked by the attB sequences were subjected to site-specific recombination with the entry vector, pDONR207 (Invitrogen), which contains the *ccdB* gene flanked by attP sites; the reactions catalyzed using BP Clonase yielded entry clones that were used to transform competent DH5a (*Escherichia coli*) cells. Using LR Clonase enzyme mix (Invitrogen), the pDONR207- *OsMADS8* clones were subjected to site-specific recombination with the plant expression vector pGWB5, which contains the cauliflower mosaic virus 35S promoter and the C-terminal GFP epitope. The resulting expression constructs (*OsMADS8-OX*) were transformed into the *Agrobacterium tumefaciens* strain GV3101 using the freeze-thaw method (Hofgen and Willmitzer, 1988).

To construct the *OsMADS8* RNAi vectors, the *OsMADS8*-specific primers were designed from the sequence of *OsMADS8*, containing the following *EcoR*I and *Xho*I restriction sites: 5' -GGATCCCAGGCAGGTGACGTTCGCG-3' and 5'-GAGCTCCTTTTATGCCAAGTGTCCC-3'. The PCR product was digested with *EcoR*I and *Xho*I and ligated into the pENTR11 vector (Invitrogen). The pENTR11*-OsMADS8* clones were then subjected to site-specific recombination with a plant RNAi expression vector, pH7GWIWG2(I), together with the cauliflower mosaic virus 35S promoter (Kim et al., 2012) using LR Clonase enzyme mix (Invitrogen). The resulting expression constructs (*OsMADS8-RNAi*) were transformed into the *A. tumefaciens* strain GV3101 using the freeze-thaw method.

The overexpression (OX) and RNA interference (RNAi) constructs were constructed and transformed into HY (hereafter *OsMADS8-*OX) and CR6078 (hereafter *OsMADS8-*RNAi), respectively, using the piercing and vacuum infiltration method (Lin et al., 2009).

### Histological Analysis

To obtain embryo sections, seeds of the three lines (HY, W1944, and CR6078) and transgenic plants were incubated in distilled water for 4 h at 4°C. Embryos were manually thin-sectioned in a petri dish using a razor blade (DORCO, Seoul, Korea). The sections (9–12 μm thick) were stained with Perls’ Prussian blue (2% hydrochloric acid mixed with 2% potassium ferrocyanide) for 10 min followed by gently washing with distilled water for 2 min. Imaging was performed with an Olympus BX40 microscope and a Carl Zeiss AxioCam MRc 5 digital camera operated by the Zeiss AxioVision AC system.

### Sequence Analysis of *DTE9* in Rice Accessions

The sequence of *DTE9* was compared among 137 Asian cultivated rice accessions (*O. sativa* L.) from the KRICE_CORE (Kim et al., 2016) and sequence variant data were provided by Kongju National University. This mini core collection is comprised of 81 *japonica*, 43 *indica*, seven aus, four admixture, and two aromatic accessions (Supplementary Table S2). To compare the *OsMADS8* sequence between W1944 and W1943, a putative ancestor of *japonica* rice (Huang et al., 2012), the 9-kb upstream and downstream of *OsMADS8* genomic sequence of W1943 was downloaded from the EnsemblPlants database.^5^

### Abscisic Acid Content Assay

The ABA content was measured using a Phytodetek^®^ ABA Kit for ABA (Agdia, Elkhart, IN, United States) according to the manufacturer’s instructions. For this ELISA-based assay, seeds were ground in liquid nitrogen, and ABA was extracted in 80% acetone for 24 h at −20°C under dark condition. The supernatant was collected after centrifugation at 13,475 *g* for 10 min and diluted 10-fold with 1× TBS (Trizma Base 3.03 g/L, NaCl 5.84 g/L, magnesium chloride hexahydrate 0.20 g/L, NaN_3_ 0.20 g/L) for use with the Phytodetek^®^ kit. The absorbance was detected at 405 nm using a Multiskan SkyHigh microplate spectrophotometer (Thermo Fisher Scientific, Waltham, MA, United States). Each assay was performed in duplicate.

### Statistical Analysis

The data were analyzed using a one-way ANOVA. Tukey’s test was used to compare the mean values. All the statistical analyses were performed using the Statistical Package for the Social Sciences (SPSS 12; SPSS Inc., Chicago, IL, United States), and statistical significance was set at either *p* < 0.05, *p* < 0.01, or *p* < 0.001.

## Results

### Characterization and Genetic Analysis of the DTE Trait

**Figure 1:**
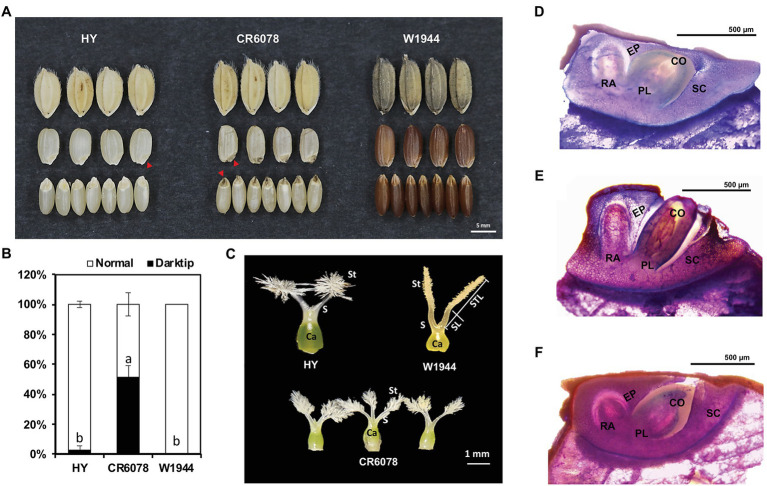
Phenotypic differences among HY, W1944, and CR6078. **(A)** Seed morphology. Red arrowhead indicates the position of dark tip embryo (DTE) in CR6078. **(B)** Comparison of the dark tip embryo rate (%). **(C)** Characterization of the pistil phenotype in HY, W1944, and CR6078. The normal pistil with one carpel (Ca) and two stigmas (St) in HY and W1944. The aberrant pistils of CR6078. One carpel with three or four stigmas. **(D–F)** Section analysis. The seed longitudinal section was stained with 0.05% toluidine blue to compare the embryo shape of HY **(D)**, CR6078 **(E)**, and W1944 **(F)**. Ca, carpel; St, stigma; S, style; CO, coleoptile; PL, plumule; RA, radicle; SC, scutellum; EP, epidermis; STL, stigma length; SL, style length.

Dark tip embryos were observed in the seeds of a BC_3_F_3_ introgression line CR6078, which originated from a cross between HY and the *O. rufipogon* accession W1944 (Figure 1A). CR6078 showed a significantly higher occurrence of DTE (approximately 42%) compared to the parental lines HY (approximately 1%) and W1944 (approximately 0%; Figure 1B). CR6078 seeds were intermediate in shape and size compared to HY and W1944 and had protruding embryos.

Differences in the number and morphology of the floral organs among the three accessions were also observed. CR6078 had one carpel with three or four stigmas, whereas HY and W1944 had normal pistils with one carpel and two stigmas (Figure 1C). We investigated the stigma characteristics of these three accessions. W1944 had longer stigmas than HY and CR6078, which is consistent with the finding that eight accessions of *O. rufipogon* and four of *O. nivara* had CC alleles at *OsSYL2* and *OsSYL3*, contributing to an increase in the stigma length in these wild *Oryza* species (Dang et al., 2020). Histological analysis of thin sections from these CR6078 embryos showed a cleavage between the coleoptile and epidermis without any tissue covering the coleoptile (Figure 1E). Cleavages were not observed and the coleoptiles were covered by the surrounding tissues in both HY and W1944 (Figures 1D,F). Another cleavage was observed between the coleoptile and scutellum in CR6078, and the scutellum on the radicle side was swollen in CR6078 (Figure 1E). These cleavages suggest that CR6078 seeds initiate a precocious germination process with coleoptile protrusion, but germination is arrested perhaps because of environmental conditions, such as water deficit in the panicle.

For genetic analysis of the DTE trait, CR6078 was crossed with HY, and the F_1_ plants (BC_4_F_1_) were self-pollinated to produce BC_4_F_2_ seeds. All the BC_4_F_2_ seeds had the HY phenotype, suggesting that DTE was present in the maternal tissue. In the BC_4_F_3_ population (204 plants), a segregation of 3 (normal): 1 (DTE) was observed, suggesting that DTE is controlled by a single recessive gene in this population from the HY/CR6078 cross (Table 1). However, the results did not explain the finding that DTE was only observed in CR6078 but not in the two parents.

**Table 1:** Genetic analysis of dark tip embryo (DTE) from two crosses.

Line^1^	Phenotype	No. of plants		*χ* ^2^	*p*
		Normal	*dte*	(3:1)/(15:1)	
HY	Normal	10	0		
W1944	Normal	10	0		
CR7277-205	*dte*	0	10		
Milyang23	Normal	10	0		
BC^3^F^3^	Normal/*dte*	153	51	0.53	*p* > 0.6
F^3^	Normal/*dte*	358	22	0.17	0.5 < *p* < 0.75

1 HY, Hwayeong; BC_3_F_3_, a cross between CR6078/HY; F_2_, a cross between CR7277-205 and Milyang23; CR7277-205, a BC_4_F_4_ line from a CR6078/HY cross with the DTE phenotype.

To better understand the genetics of DTE, a second cross was made between an *indica* rice cultivar Milyang23 and CR6078. A total of 370 F_2_ plants were grown, and F_3_ seeds were obtained from each F_2_ plant. All the F_2_ seeds were Milyang23 type, and the F_3_ population segregated 347 normal and 23 DTE plants, suggesting that DTE was controlled by two recessive complementary genes (Table 1). When more than 300 F_3_ plants from the cross between HY and Milyang23 were screened, no F_3_ line with the DTE phenotype higher than 1% was detected. The results suggested that two or more non-allelic genes regulate the DTE trait, which is not detected in the parents, and that the W1944 allele is required to display DTE.

The pollen viability of HY and CR6078 was also evaluated. The pollen grains of HY and CR6078 were uniform in size and shape and approximately 90% were viable, indicating that the low fertility was not associated with pollen viability of CR6078 (Supplementary Figure S4).

### Construction of a High-Density Map and Identification of *DTE* Candidate Genes

**Figure 2:**
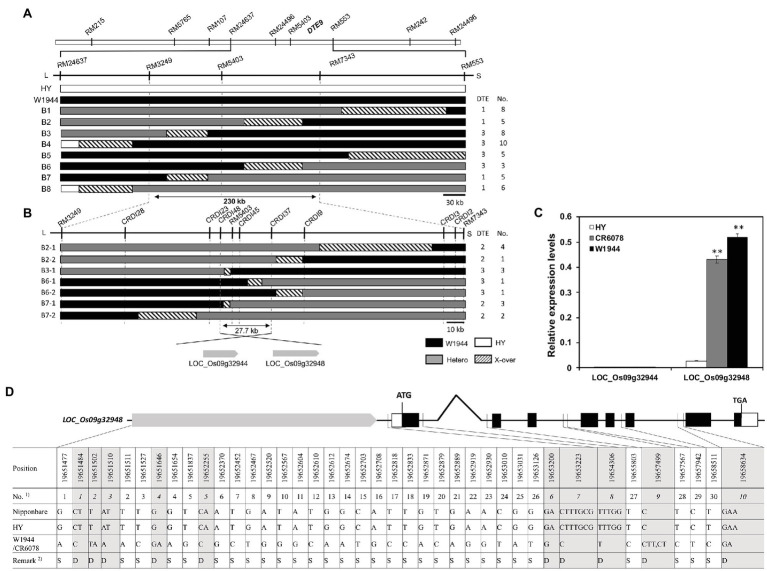
Graphical genotypes and substitution mapping of *DTE9*. **(A)** Substitution mapping of *DTE9*^OR^ using F_5_ lines. Comparison of the genotypes of lines delimiting *DTE9*^OR^ between the markers RM3249 and RM7343. Physical distance is about 230-kb based on the Nipponbare sequence. **(B)** High-density mapping of *DTE9*. Comparison of the genotypic recombinants delimiting *DTE9* between the markers CRDI37 and CRDI48. White, black, and gray bars indicate homozygous and heterozygous alleles for HY and W1944 and hatched areas indicate regions where the crossing-over occurred on chromosome 9. Two genes were predicted in the target 27.7 kb region. **(C)** Expression levels of the *DTE9* candidate genes in the three cultivars. Total RNA was isolated from 7-day-old seedlings of the three lines. Relative expression level of each gene was quantified using real-time PCR and normalized using *OsTMP1* as an internal control. The relative transcript levels of *DTE9-*involved candidate genes (LOC_Os09g32944, LOC_Os09g32948). The significant differences between the HY and CR6078 plants and the HY and W1944 at ^**^p < 0.01. **(D)** Comparison of promoter and genomic sequence of *OsMASD8* in HY and W1944. A total of 30 single nucleotide polymorphisms (SNPs) and 10 InDels was identified between HY and W1944. ^1)^SNP and InDel are separately numbered in bold and italics, respectively. ^2)^S: SNP, D: InDel.

Fifteen SSR markers on the chromosomes 1, 2, 5, 7, 8, 9, and 11 detected W1944 segments in CR6078 (Supplementary Figure S2). A BC_4_F_3_ population of 204 individuals derived from a cross between HY and CR6078 was evaluated for DTE and some agronomic traits, such as TGW (g), RR (%), and GW (g) per plant. The frequency distribution of the DTE trait in the BC_4_F_3_ population followed a Mendelian ratio of 3 normal:1 DTE (*χ*^2^ = 0.53, 0.1 < *p* < 0.5), indicating that this trait is controlled by a single recessive gene. The gene was mapped to the interval between RM553 and RM24637 on chromosome 9 and designated *DTE9* (Figure 2A). The frequency distribution of TGW, RR, and GW per plant, also followed a 3:1 segregation ratio (Table 2). The single-point analysis showed that the QTL for all four traits were linked to *DTE9*. These results suggest that *DTE9*^OR^ (*O. rufipogon* W1944 allele) has a pleiotropic effect (Table 2).

**Table 2:** Quantitative trait loci (QTL) for the three traits based on a single-point analysis in the F_3_ population.

Traits^1^	QTL	Pop.	*p*	*R* ^2^	Phenotypic mean^2^		
					HH	HW	WW
GW (g)	*gw9*	F^3^	<0.001	5.0	35.2 ± 9.2a^3^	38.6 ± 13.4a	27.5 ± 9.7b
RR (%)	*rr9*	F^3^	<0.001	46.6	94.9 ± 2.2a	94.3 ± 2.1a	75.1 ± 9.8b
TGW (g)	*tgw9*	F^3^	<0.001	36.6	25.4 ± 0.8a	25.8 ± 1.3a	22.2 ± 1.1b

1 GW, grain weight per plant; RR, Ripening ratio; TGW, 1,000-grain weight.

2 HH, Hwayeong homozygote; HW, Hwayeong/W1944 heterozygote; WW, W1944 homozygote.

3 Numbers followed by the same letter are not significantly different at *p* < 0.05 by Tukey’s test.

For fine mapping of *DTE9*, six heterozygous plants at RM553 and RM24637 were selected from the BC_4_F_3_ population to produce a BC_4_F_4_ population, which was screened with the SSR markers RM553 and RM24637 to identify recombinants. Substitution mapping using 50 BC_4_F_4_ recombinants indicated that *DTE9*^OR^ was located between the RM7343 and RM3249. This 230-kb interval has 38 genes based on the Nipponbare reference genome (Figure 2A). Sixty insertion/deletion (InDel) markers were designed by comparing the Nipponbare sequences of 38 genes with that of 93-11 sequences. Eight markers showed polymorphisms between HY and W1944 (Supplementary Table S1). Genotyping of 15 recombinants with eight InDel markers delimited *DTE9*^OR^ to a 27.7 kb interval flanked by markers CRDI48 and CRDI37. This interval contains two predicted genes, LOC_Os09g32944 and LOC_Os09g32948 (Figure 2B). LOC_Os09g32944 encodes the SQUAMOSA promoter-binding-like protein 18 (*OsSPL18*), which controls ligule development, grain weight, and grain number in rice (Lee et al., 2007b; Yuan et al., 2019). LOC_Os09g32948 encodes *OsMADS8/24*, a member of the MADS-box transcription factor gene family in rice, which is involved in floral organ specification, plant growth, and development (Arora et al., 2007).

### *DTE9* Candidate Gene Analysis

The expression of the two candidate genes was compared among HY, W1944, and CR6078 (Figure 2C). No differences in *OsSPL18* expression were observed among the three lines. In contrast, *OsMADS8* was expressed at higher levels in both CR6078 and W1944 compared to HY suggesting that *OsMADS8* is more likely the candidate gene for *DTE9*^OR^ (Figure 2C). Thirty single nucleotide polymorphisms (SNPs; 26 and four in the promoter and intron regions, respectively) and 10 InDels (seven and three in the promoter and intron regions, respectively) were detected in the *OsMADS8* sequences between HY and W1944 (Supplementary Table S2). The lack of sequence differences in the eight exon regions suggests that the variation in the *OsMADS8* promoter region underlies the differential expression of this gene in HY and W1944/CR6078.

### Genetic Diversity Analysis of *OsMADS8*

Forty *OsMADS8* sequence variants between HY and W1944 were detected in the promoter and intron regions. Two hotspots were observed in the promoter region between the nucleotides 19,651,477 and 19,651,511 (SNP1-2 and InDel1-3) and between nucleotides 19,653,010 and 19,654,306 (SNP24-26 and InDel6-8; Figure 2D). Considering that the promoter is responsible for regulating the expression of *OsMADS8*, the difference between the two variable promoter regions might be associated with the variation of DTE trait in CR6078.

When the comparison was expanded to 137 accessions from the KRICE_CORE collection, a total of 81 SNPs and 35 InDels were detected in the promoter, 5' UTR, and intron regions of *OsMADS8* (Supplementary Tables S3–S6). Eighteen out of 40 SNPs in the promoter and intergenic regions were *indica* or *japonic*a-specific (Supplementary Table S5). Among these subspecies-specific SNPs, W1944 had different SNPs from those of W1943, a putative ancestor of *japonica* rice (Huang et al., 2012). For SNP5, W1944 with an A-allele was different from all 137 accessions with a G-allele. In addition, 14 and 21 InDels were observed in the promoter and intron regions, respectively (Supplementary Table S4). Ten InDels were observed in the 137 accessions and half of the 10 InDels were species-specific. Six InDels were W1944-specific compared to W1943 (Supplementary Table S6).

### *DTE9* Is an Allele of *OsMADS8*

**Figure 3:**
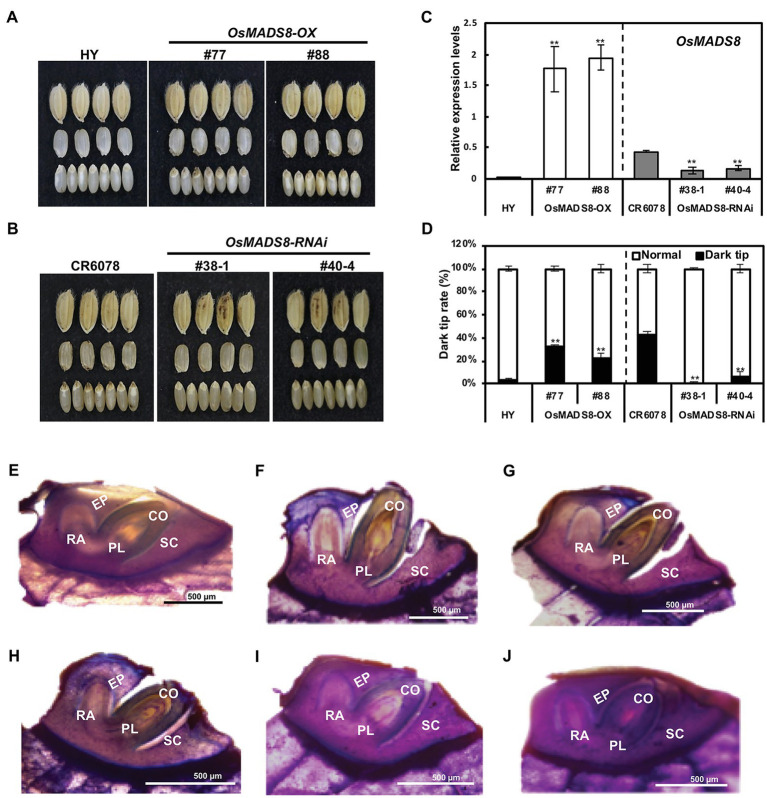
Characterization of the *OsMADS8* transgenic lines. **(A)** Comparison of the grain between HY and *OsMADS8*-OX (#77, #88) lines. **(B)** Comparison of the grain between CR6078 and *OsMADS8*-RNAi (#38–1, #40–4) lines. **(C)** The expression level of the *OsMADS8* gene in the transgenic plants. **(D)** Dark tip embryo rate (%). Histological analysis of the embryo shape of HY **(E)** and two OX lines **(F,G)** and CR6078 **(H)** and *OsMADS8*-RNAi lines **(I,J)**. SC, scutellum; CO, coleoptile; PL, plumule; EP, epiblast; and RA, radicle. ** indicates significant difference based on the student’s *t*-test at *p* < 0.01.

To confirm the relationship between *OsMADS8* and the abnormal embryo phenotype (DTE), the W1944 full-length *OsMADS8* open reading frame was expressed under the control of the cauliflower mosaic virus 35S promoter. Overexpression (OX) and knockdown (RNAi) constructs were developed and transformed into HY (hereafter *OsMADS8-*OX) and CR6078 (hereafter *OsMADS8-*RNAi), respectively. Six independent *OsMADS8-*OX lines and 46 independent *OsMADS8-*RNAi lines were generated (Figures 3A,B). The T_2_ plants of *OsMADS8-*OX lines #77 and #88 showed significantly higher expression of *OsMADS8*, whereas T_2_ plants of *OsMADS8-*RNAi lines #38-1 and #40-4 showed significantly reduced transcript levels of *OsMADS8* (Figures 3C,D). Significant differences between HY and *OsMADS8-*OX and CR6078 and *OsMADS8-*RNAi were observed for grain weight and ripening ratio (i.e., fertility), but not for the other traits (Supplementary Table S7). When the seeds of HY and *OsMADS8-*OX were compared, OX lines showed a higher rate of occurrence of DTE (about 22–33%) than HY (~3%). As expected, the two RNAi lines had lower DTE rates (~0.2%) than CR6078 (~42%; Figure 3D). Taken together, these results indicate that *OsMADS8* is associated with the DTE phenotype (Figures 3A–D). The histological analysis showed that the coleoptile, plumule, and radicle of *OsMADS8-*OX embryos were protruded, showing clear cleavage from the scutellum tissue (Figures 3E–G). In contrast, the embryos of the HY and the *OsMADS8-*RNAi lines had coleoptiles embedded in the endosperm and appeared normal (Figures 3H–J). These results indicated that *DTE9* is an allele of *OsMADS8*.

### Expression of ABCDE Floral Organ Identity Genes

**Figure 4:**
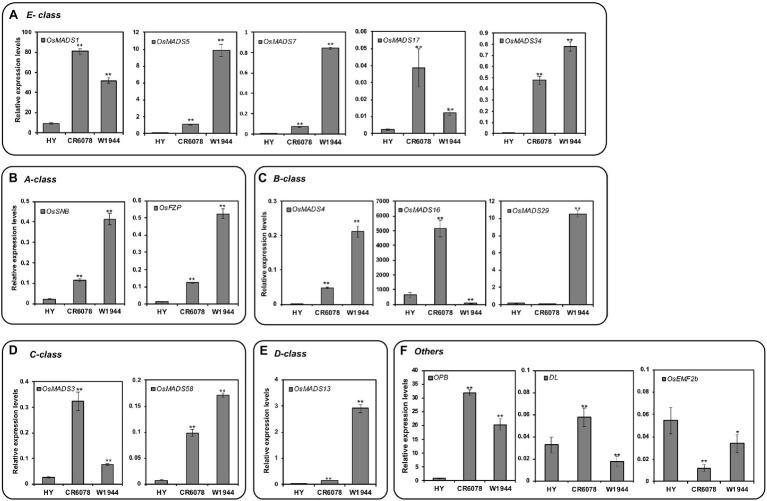
Expression patterns of floral organ determination genes in HY, CR6078, and W1944 plants. **(A)** E-class, **(B)** A-class, **(C)** B-class. **(D)** C-class, **(E)** D-class genes, and **(F)** other genes. Total RNA was isolated from 7-day-old seedlings. Relative expression level of each gene was quantified using real-time PCR and normalized using *OsTMP1* as an internal control. The error bars represent mean ± SD (*n* = 3). Significant differences between the HY and each OX line, and the CR6078 plants and each RNAi line at ^*^p < 0.05 and ^**^p < 0.01.

CR6078 has more styles and stigmas than HY and W1944, indicating that the floral organ identity genes were differentially regulated in this line (Figure 1C). Therefore, the expression of genes possibly interacting with *OsMADS8,* an E-class floral identity group gene, was analyzed. Among five other E-class genes, the expression of *OsMADS5*, *OsMADS7*, and *OsMADS34* was the highest in W1944, followed by CR6078 and HY (Figure 4A; Supplementary Figure S1). The higher expression of these three genes in CR6078 compared to HY suggests a background effect from W1944 in CR6078. Interestingly, CR6078 showed higher expression of *OsMADS1* and *OsMADS17* than HY and W1944, indicating that *OsMADS8* might regulate *OsMADS1* and *OsMADS17* expression. This finding is consistent with the DTE trait in CR6078 resulting from the combined effect of the two complementary genes, the W1944 allele of *OsMADS8* and an unknown gene from HY (Figure 4A).

To examine potential interactions with other floral homeotic genes, we analyzed the expression of the ABCD group genes. *OsSNB*, *OsMADS4*, and *OsFZP* were expressed at higher levels in W1944 than in CR6078 and HY (Figures 4B,C). It is noteworthy that *OsMADS16* was expressed at higher levels in CR6078 than in HY and W1944, whereas *OsMADS29* was expressed at a lower level in CR6078 than in HY and W1944 (Figure 4C). The expression of the C-class gene *OsMADS3* was remarkably higher in CR6078 than in HY and W1944 (Figure 4D).

**Figure 5:**
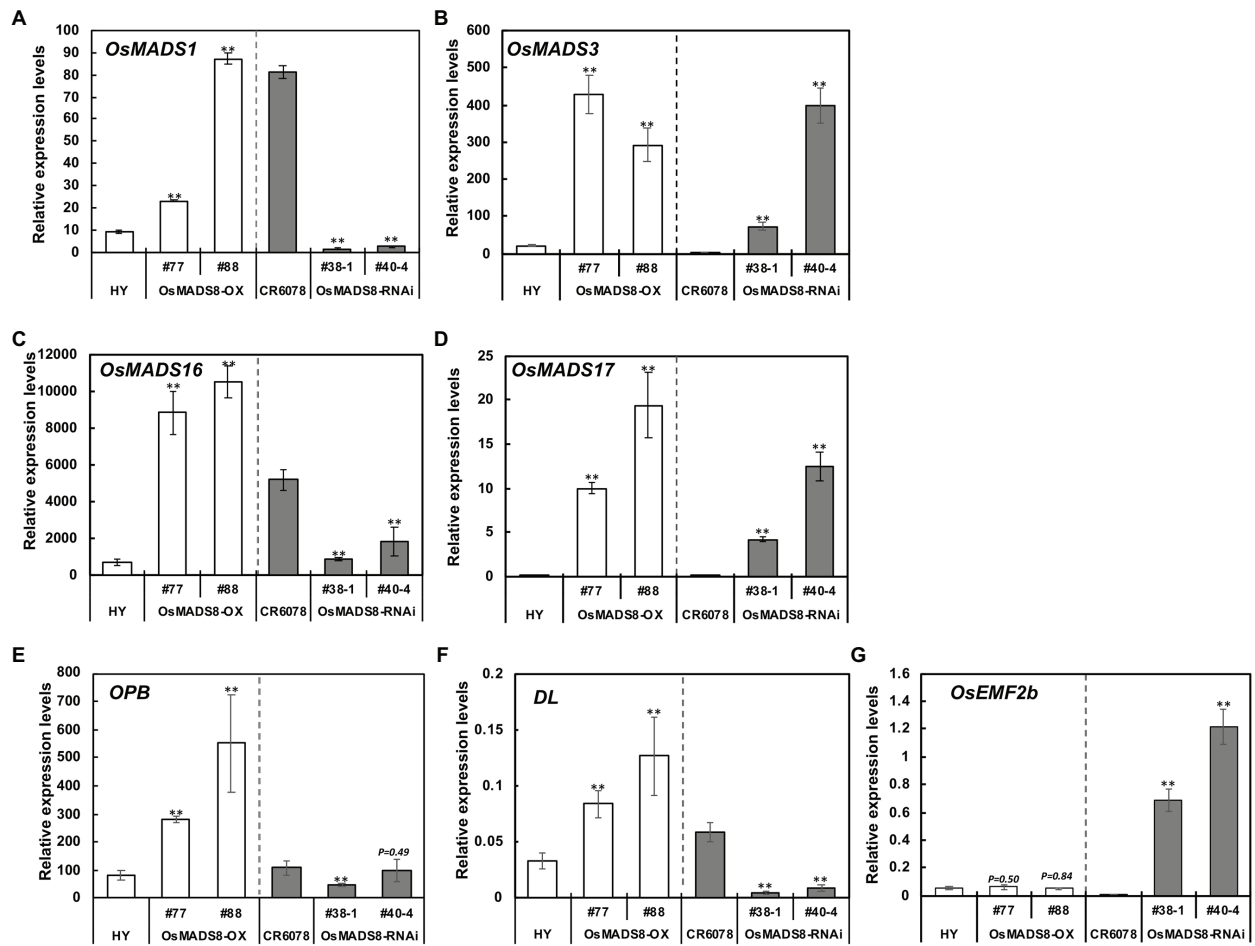
Expression of seven floral development genes in the *OsMADS8* transgenic lines. **(A)** *OsMADS1*, **(B)** *OsMADS3*, **(C)** *OsMADS16*, **(D)** *OsMADS17*, **(E)** *OPB*, **(F)** *DL*, and **(G)** *OsEMF2b*. Total RNA was isolated from the 7-day-old seedlings of the *OsMADS8* transgenic lines. Relative expression level of each gene was quantified using real-time PCR and normalized using *OsTMP1* as an internal control. The error bars represent mean ± SD (*n* = 3). The significant differences between the HY and each OX line and the CR6078 plants and each RNAi line were evident at ^**^p < 0.01.

The floral organ identity genes that exhibited higher or lower expression in CR6078 compared to the parental lines HY and W1944 were then investigated in the transgenic plants (Figure 5). The expression of these four genes, *OsMADS1*, *3*, *16*, and *17*, was higher in *OsMADS8*-OX than in HY. The *OsMADS8*-RNAi lines also showed lower expression of *OsMADS1* and *OsMADS16* than in CR6078. Unexpectedly, the *OsMADS8*-RNAi lines showed higher expression of *OsMADS3* and *OsMADS17* than CR6078 (Figures 5B,D). These results indicate that *OsMADS8* directly or indirectly regulates the expression of these genes, which may underlie the formation of the abnormal floral structure including pistil in CR6078.

Expression of the other ABCDE functional floral identity group genes was also examined in the transgenic lines. Among the A- and B-class genes, *OsSNB* and *OsFZP* exhibited higher expression in the *OsMADS8-*OX lines than in HY. The expression of *OsSNB* and *OsFZP* was at higher level in the *OsMADS8*-RNAi lines compared to CR6078, however, unexpectedly *OsMADS4* and *OsMADS29* showed lower expression levels in CR6078 than in the *OsMADS8*-RNAi lines (Supplementary Figures S5A–D). Among the C-class genes, *OsMADS58* was expressed at higher levels both in the OX and RNAi transgenic lines, whereas the expression of *OsMADS13* was remarkably higher in the *OsMADS8*-OX lines than in HY (Supplementary Figures S5E,F). Among the E-class genes, *OsMADS5* and *OsMADS34* expression was remarkably higher in the *OsMADS8*-OX lines than in HY (Supplementary Figures S5G,H).

No differences in the number of stigmas and styles were observed between HY and *OsMADS8*-OX lines (Supplementary Figure S6). In addition, the *OsMADS8*-RNAi lines did not show any difference in the number of floral organs compared to CR6078 plants (Supplementary Table S8; Supplementary Figure S7). These results suggest that the *OsMADS8* expression in the *OsMADS8*-OX and *OsMADS8*-RNAi lines was not sufficient to induce phenotypic changes in the floral organs.

### *OsMADS8* Regulates the Expression of Other Floral Determination Genes

Previous studies have shown that other genes are also involved in determining the carpel and style or stigma identity (Cui et al., 2010; Li et al., 2011; Conrad et al., 2014; Deng et al., 2017; Zhang et al., 2018). Interestingly, the transcription levels of *Open Beak* (*OPB*) and *Drooping Leaf* (*DL*) in CR6078 were higher than those in HY and W1944 (Figure 4F). In contrast, *OsEMF2b* was expressed at lower levels in CR6078 than in HY and W1944. In the *OsMADS8-*OX plants, the transcript levels of *OPB* and *DL* were higher than those in HY, whereas the expression of *OsEMF2b* was not significantly different (Figures 5E–G). As expected, the transcript levels of *OPB* and *DL* in the *OsMADS8*-RNAi plants were lower than those in CR6078, whereas the *OsEMF2b* expression was higher (Figures 5E–G). Thus, *OsMADS8* in CR6078 altered the expression of *OPB*, *DL*, and *OsEMF2b* in addition to the homeotic genes of the ABCDE model of flower development.

### *OsMADS8* Negatively Regulates the Expression of *OsEMF2b* in the ABA Signaling Pathway

**Figure 6:**
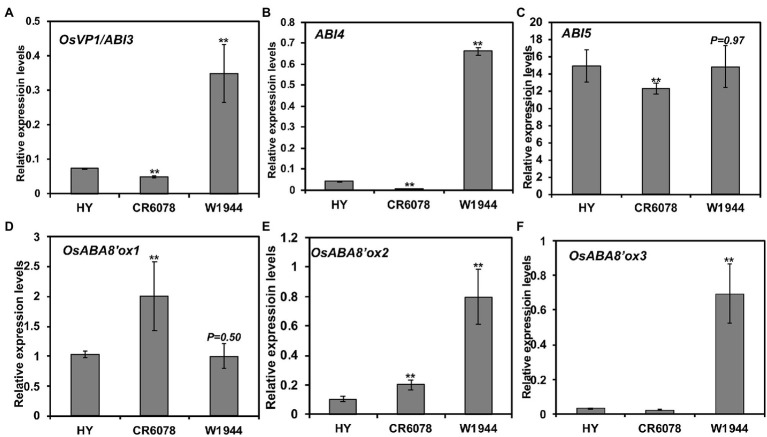
Expression of abscisic acid (ABA) signal transduction and ABA catabolism-related genes in three accessions **(A–F)**. Seeds from the three lines were used to isolate the total RNA. Relative expression level of each gene was quantified using real-time PCR and normalized using *OsTMP1* as an internal control. The error bars represent mean ± SD (*n* = 3). The significant differences between the HY and each OX line and the CR6078 plants and each RNAi line were evident at ^**^p < 0.01.

To explain the DTE trait exhibited by CR6078, *OsEMF2b* was investigated based on the previous finding that this gene directly regulates the expression of *OsVP1/ABI3* and the ABA signaling genes regulating seed germination (Chen et al., 2017). CR6078 displayed reduced expression levels of *OsVP1/ABI3, ABI4*, and *ABI5* compared to that of HY and W1944 (Figures 6A–C). Among the three genes regulating ABA catabolism, the *OsABA8’ox1* expression levels were higher in CR6078 than in HY and W1944, while no clear pattern was observed in the expression of *OsABA8’ox2* and *OsABA8’ox3* (Figures 6D–F), suggesting that DTE might be associated with altered expression of *OsEMF2b* and *OsABA8’ox1,* which regulates seed dormancy and germination.

To determine whether *OsEMF2b* is associated with changes in embryonic traits, the expression of genes regulating the ABA signaling pathway and catabolism in *OsMADS8*-OX and RNAi plants was examined. The expression of *ABI3* and *ABI4* was significantly reduced in *OsMADS8*-OX compared to that of HY (Figure 6). However, the expression of *ABI5* was significantly reduced in HY compared to that in *OsMADS8*-OX (Supplementary Figures S8A–C). All three genes (*OsABA8’ox1*, *OsABA8’ox2*, and *OsABA8’ox3*) related to ABA catabolism showed higher expression in the *OsMADS8*-OX plants than in HY (Supplementary Figures S8D–F). The expression of *ABI3*, *ABI4*, and *ABI5* was significantly increased in *OsMADS8*-RNAi compared to that of CR6078 (Supplementary Figures S8A–C). By contrast, the expression of *OsABA8’ox1* was lower in the *OsMADS8*-RNAi plants than in CR6078, whereas *OsABA8’ox3* was expressed at a higher level in the *OsMADS8*-RNAi than in CR6078 (Figures 6D–F). Taken together, these results suggest that *OsMADS8* negatively regulates the expression of *OsEMF2b*, a positive regulator of seed dormancy, by overexpressing *OsABA8’ox1*.

**Figure 7:**
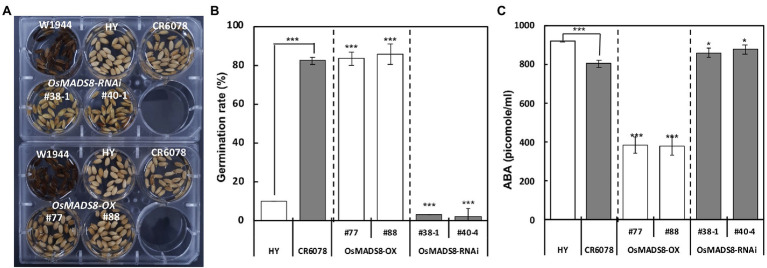
Comparison of germination ratio at 30°C and ABA contents in the transgenic and control plants. **(A)** The CR6078 and *OsMADS8*-OX lines showed a significantly higher germination ratio than the HY and *OsMADS8*-RNAi at 24 h after sowing. **(B)** The significant differences between the HY and CR6078, the HY and each OX line, and the CR6078 plants and each RNAi line were evident at ^***^p < 0.001. The germination rate (based on the length of radicles >1 mm) was recorded, and values are represented as mean ± SD (*n* = 3). **(C)** Analysis of the ABA content in the *OsMADS8* transgenic lines and controls. The error bars represent mean ± SD (*n* = 3). Data represent the average of three replicates. The comparison was made between the HY and CR6078, and the HY and each OX line, and the CR6078 and each RNAi line. Significant differences were evident at ^*^p < 0.05 and ^***^p < 0.001.

To determine the effect of *OsMADS8* on germination, the germination ratio of HY, CR6078, W1944, and the transgenic lines were compared. CR6078 and the *OsMADS8*-OX lines started germination at 24 h after sowing with a germination ratio of 82% while the germination ratios of HY and *OsMADS8*-RNAi lines were less than 3% (Figures 7A,B). The ABA content in the seeds of HY, CR6078, and transgenic lines was analyzed to determine whether the decreased germination ratio was related to the ABA content. The ABA level of *OsMADS8*-OX plants (385–377 pmoles ml^−1^) was significantly lower than that of HY (919 pmoles ml^−1^; Figure 7C). The two *OsMADS8*-RNAi lines had significantly higher ABA contents than CR6078 (Figure 7C). These results support the idea that in the CR6078 line, *OsMADS8* regulates the expression of genes involved in ABA catabolism (Figure 7).

## Discussion

### *DTE9* Is a Novel Gene Associated With Hybrid Weakness

Hybrid weakness, the poor development of hybrids compared to their parents, hinders gene exchange between different species at the postzygotic stage. Hybrid weakness has been reported in rice at both the interspecific or intraspecific crosses, and most studies have reported that this phenomenon is mainly controlled by dominant or recessive complementary interactions of unlinked loci (Fukuoka et al., 1998, 2005; Kubo and Yoshimura, 2002; Matsubara et al., 2007; Yamamoto et al., 2007; Jiang et al., 2008). Characterization of the genetic basis of hybrid weakness contributes to our knowledge of the underlying mechanisms of reproductive isolation and has practical significance with regard to the maximal utilization of invaluable genes from the interspecific or intraspecific crosses in plant breeding (Chen et al., 2014). This study characterized a newly discovered gene for hybrid weakness in rice, *DTE9*. The DTE trait was observed in CR6078, an introgression line derived from an interspecific cross between *O. sativa* spp. *japonica* “Hwayeong” and the wild relative *O. rufipogon*.

To understand the genetic basis of DTE, a population derived from a cross between HY and CR6078 was developed. In the BC_4_F_3_ population, a segregation of 3 (normal): 1 (DTE) was observed, suggesting that DTE is controlled by a single recessive gene in this cross. However, a single recessive gene mode of inheritance for DTE is not compatible with the finding that the two parental lines do not display DTE. The F_3_ population from a second cross between an *indica* rice cultivar Milyang23 and CR6078 displayed a segregation ratio of 15:1 (normal:DTE). These results support the involvement of two complementary recessive genes, which is consistent with the previous findings that two complementary recessive genes regulate hybrid weakness in rice (Sato, 1997; Fukuoka et al., 1998; Kubo and Yoshimura, 2002; Ichitani et al., 2007; Matsubara et al., 2007; Kuboyama et al., 2009). Based on our genetic analysis of the progeny from two crosses, we hypothesize that two or more genes (QTLs), *OsMADS8* and other unknown genetic factors control DTE in rice (Table 2). Another interesting feature of DTE was that all F_2_ seeds from the F_1_ plants of the two crosses showed normal Milyang23 and HY embryo types with segregation observed in the F_3_ population. This suggests that the DTE trait is of maternal tissue origin.

Dark tip embryo was significantly associated with several deleterious traits including reduced GW (Table 2). Considering that there are no reports or annotations for traits of agronomic importance in or near this region, *DTE9* is likely to be associated with the deleterious phenotypes. Based on the DTE phenotype, it is possible that the CR6078 seeds, which have significantly lower ABA content than that of HY, began preharvest germination with coleoptile protrusion. However, germination was arrested perhaps due to environmental conditions, such as water deficit in the panicle. During the germination process, CR6078 consumed carbohydrates in grains, leading to a decrease in the GW. Given the association of *DTE9* with several deleterious traits, the interaction between *OsMADS8* and the other gene would likely not be selected for during breeding and domestication.

### Changes in Floral Organs of CR6078 Is Caused by Various Floral Identity Genes

CR6078 displayed an abnormal floral organ phenotype with more styles and stigmas than those of HY and W1944. Gene expression analyses showed that the alteration of *OsMADS8* expression in CR6078 and transgenic plants not only changed the expression of the various ABCDE model genes, but also that of genes, such as *OPB* and *OsEMF2b* (Figure 4). It is noteworthy that among the ABCDE model genes, the expression of *OsMADS1, 3, 16*, and *17* was higher in CR6078 than in HY and W1944. Additionally, *OPB* and *DL* displayed similar expression patterns, whereas CR6078 showed lower expression of *OsEMF2b* than HY and W1944. A similar expression pattern was also observed for the genes associated with the ABA signaling pathway and catabolism in CR6078, supporting the idea that the two complementary genes, *OsMADS8*, and another unknown gene in HY, control the DTE in this population (Figure 6). However, the *OsMADS8*-RNAi lines showed higher expression of *OsMADS3* and *OsMADS17* than CR6078 (Figure 5). The expression patterns of several genes were not the same in the transgenic plants as in CR6078. For example, *OsMADS*3 and *OsMADS17* showed higher expression in the *OsMADS8*-RNAi lines than in CR6078, and *ABI5* and *OsABA8’ox3* displayed higher expression in the *OsMADS8*-OX lines than in HY. This is probably due to interactions between transgenes and genes associated with the floral organ phenotype in the background of HY and CR6078. Moreover, CR6078 harbors eight *O. rufipogon* segments in the HY background.

*OsMADS8*-OX plants failed to show phenotypic changes compared to HY, possibly owing to the low expression of *OsEMF2b* in the *OsMADS8*-OX lines. In a previous study, the loss of function of *EMF2b* resulted in abnormal changes in the internal floral organ structure. When the phenotype of the *emf2b* mutant was compared with that of CR6078, the *emf2b* mutant showed more carpels and stigmas and had lower expression of genes including *OsMADS1*, *OPB*, and *DL* than the wild type (Conrad et al., 2014; Deng et al., 2017). In contrast, CR6078 showed higher expression of *OsMADS1*, *OPB*, and *DL* and lower expression of *OsEMF2b* than HY, leading to phenotypic changes in CR6078. These results raise the possibility that *OsMADS8* directly or indirectly regulates the expression of the B-, E-, and C-class genes as well as other genes, such as *OPB* and *OsEMF2b*, and this regulation is responsible for the changes in the inner floral organ phenotype observed in CR6078. It is rational to infer that *OsMADS8* alone induces a weak phenotypic change (Cui et al., 2010).

### *OsMADS8* Negatively Regulates the Expression of *OsEMF2b* in the ABA Signaling Pathway

*OsEMF2b* regulates seed dormancy and seedling growth by activating and inhibiting the expression of the ABA signaling response genes (Chen et al., 2017). ABA promotes seed dormancy and suppresses seed germination and seedling growth. Downregulation of *OsEMF2b* leads to vivipary and decreased expression levels of *OsVP1*, an ortholog of *ABI3*, which is a component of the ABA signaling pathway in *Arabidopsis*. CR6078 displayed reduced expression levels of *OsEMF2b*, *OsVP1*/*ABI3*, *ABI4*, and *ABI5* compared to that of HY and W1944 (Figures 4F, 6). Additionally, the expression of genes related to ABA catabolism was differentially regulated in CR6078 (Figures 6D–F). Expression of these genes was examined in the *OsMADS8*-OX and RNAi transgenic plants (Supplementary Figure S8), revealing that *OsMADS8* negatively regulates the expression of *OsEMF2b*, which is related to the activation and repression of expression of the ABA signal responsive genes. Downregulation of *OsEMF2b* expression might be associated with vivipary and decreased expression levels of *OsVP1*, which is related to the seed ABA signaling pathway and seed dormancy. Taken together, our results indicate that *OsMADS8* plays an important role in regulating the expression of *OsEMF2b* associated with floral organ determination and seed germination *via* the ABA signaling pathway and ABA catabolism.

Several studies have reported the role of plant hormones in hybrid weakness (Reiber and Neuman, 1999; Yamada et al., 2001; Yamada and Marubashi, 2003; Hannah et al., 2007; Kobori et al., 2007). Our finding that HY had significantly higher seed ABA content than CR6078 is consistent with the role of *OsMADS8* in controlling DTE in CR6078 by directly influencing the expression of genes regulating ABA catabolism. Thus, the hybrid weakness exhibited by CR6078 underscores the importance of plant hormones in this phenomenon (Yamada et al., 2001; Kobori et al., 2007).

In this study, a novel interspecific hybrid weakness gene controlling DTE phenotype was discovered in an introgression line derived from a cross between the *japonica* variety “Hwayeong” and the common wild rice W1944 (*O. rufipogon*). We characterized the phenotypic and physiological properties of this newly discovered expression of interspecific hybrid weakness and isolated the causal gene, *DTE9*, responsible for the DTE and several other traits of hybrid weakness. Based on the finding that two recessive complementary genes are responsible for full expression of the DTE trait, further studies to identify this second gene are in progress. These results contribute to a better understanding of the molecular mechanism causing hybrid weakness and will benefit rice breeding programs by assisting in strategies to maximize exploitation of invaluable genes from the interspecific or intraspecific crosses.

## Data Availability Statement

The original contributions presented in the study are included in the article/Supplementary Material, further inquiries can be directed to the corresponding author.

## Author Contributions

SK, S-DJ, and S-NA designed the experiments and wrote the manuscript. TT edited the manuscript and provided advice on the experiments. H-SL, K-CS, CA, and NL conducted the agronomic traits investigation and qRT-PCR analysis. S-DJ, PY, and H-JK performed fine mapping of *DTE9*. Y-AJ and SK analyzed the sequencing data. All authors contributed to the article and approved the submitted version.

## Funding

This work was carried out with the support of “Cooperative Research Program for Agriculture Science and Technology Development (Project No. PJ015757)” Rural Development Administration, Republic of Korea.

## Conflict of Interest

H-JK was employed by the company LG Chem., Ltd.

The remaining authors declare that the research was conducted in the absence of any commercial or financial relationships that could be construed as a potential conflict of interest.

## Publisher’s Note

All claims expressed in this article are solely those of the authors and do not necessarily represent those of their affiliated organizations, or those of the publisher, the editors and the reviewers. Any product that may be evaluated in this article, or claim that may be made by its manufacturer, is not guaranteed or endorsed by the publisher.

## Footnotes

^1^ http://www.bioneer.co.kr

^2^ http://www.gramene.org

^3^ https://plants.ensembl.org/Oryza_indica/Info/Index

^4^ http://rapdb.dna.affrc.go.jp

^5^ https://plants.ensembl.org/index.html
