# Supplementary material for: A Novel Embryo Phenotype Associated With Interspecific Hybrid Weakness in Rice Is Controlled by the MADS-Domain Transcription Factor OsMADS8
Source: Front Plant Sci. 2022 Jan 5;12:778008. doi: 10.3389/fpls.2021.778008 (PMC8769243; doi:10.3389/fpls.2021.778008)
Supplement: Supplementary file 1 [file Data_Sheet_1.PDF]

<table>
<tr><th colspan="3">Palea</th><th colspan="3">Pistil</th><th></th><th></th></tr>
<tr><th>Le</th><th>Bop Mrp</th><th>Lo</th><th>St</th><th>Ca</th><th>Ov/FD</th><th>Class</th><th>References</th></tr>
<tr><td>DL</td><td></td><td></td><td></td><td>DL</td><td></td><td rowspan="3">Others</td><td rowspan="3">Nagasawa et al., 2003; Ishikawa et al., 2009; Li et al., 2011; Horigome et al., 2009; Xiao et al., 2009; Deng et al., 2017; Conrad et al.,2014; Hannah et al., 2007; Murai et al., 2013</td></tr>
<tr><td colspan="6">OPB, OsEMF2b</td></tr>
<tr><td></td><td></td><td colspan="4">OsAPO1</td></tr>
<tr><td colspan="5">SNB, OsMADS14. OsMADS15</td><td></td><td rowspan="2">A-class</td><td rowspan="2">Murai et al., 2013; Angenent and Colombo 1996; Pelaz et al., 2000; Komatsu et al., 2003; Lee et al., 2007; Bai et al., 2016; Pasriga et al., 2019; Becker et al., 2000; Callens et al., 2018; Coen et al., 1991</td></tr>
<tr><td>FZP</td><td></td><td></td><td>FZP</td><td></td><td></td></tr>
<tr><td></td><td></td><td colspan="2">OsMADS2, OsMADS4, OsMADS16</td><td></td><td></td><td>B-class</td><td>Murai et al., 2013; Coen et al., 1991; Callens et al., 2018; Lee et al., 2003</td></tr>
<tr><td></td><td></td><td></td><td colspan="3">OsMADS3, OsMADS58</td><td>C-class</td><td>Murai et al., 2013; Coen et al., 1991; Pelaz et al., 2000; Song et al., 2018; Li et al., 2011</td></tr>
<tr><td></td><td></td><td></td><td colspan="2">OsMADS21</td><td>OsMADS13, OsMADS21</td><td>D-class</td><td>Murai et al., 2013; Coen et al., 1991; Callens et al., 2018</td></tr>
<tr><td></td><td colspan="2">OsMADS6, OsMADS17</td><td></td><td></td><td>OsMADS6, OsMADS17</td><td rowspan="4">E-class</td><td rowspan="4">Murai et al., 2013; Arora et al., 2007 ; Jeon et al., 2000; Kang et al., 1997; Zahn et al.,2005; Callens et al., 2018; Agrawal et al., 2005; Prasad et al., 2005; Conrad et al.,2 014; Cui et al., 2010</td></tr>
<tr><td></td><td></td><td colspan="4">OsMADS7, OsMADS8</td></tr>
<tr><td colspan="2">OsMADS1</td><td></td><td></td><td colspan="2">OsMADS1</td></tr>
<tr><td colspan="6">OsMADS5, OsMADS34</td></tr>
<tr><td colspan="8">Bop, body of palea; Ca, carpel; FD, floral meristem determinacy; Le, lemma; Lo, lodicule; Mrp, marginal region of palea; Ov, ovule; Pa, palea; Pi, pistil; St, stamen</td></tr>
</table>

Supplementary Figure 1. List of genes involved in floral organ determination in rice

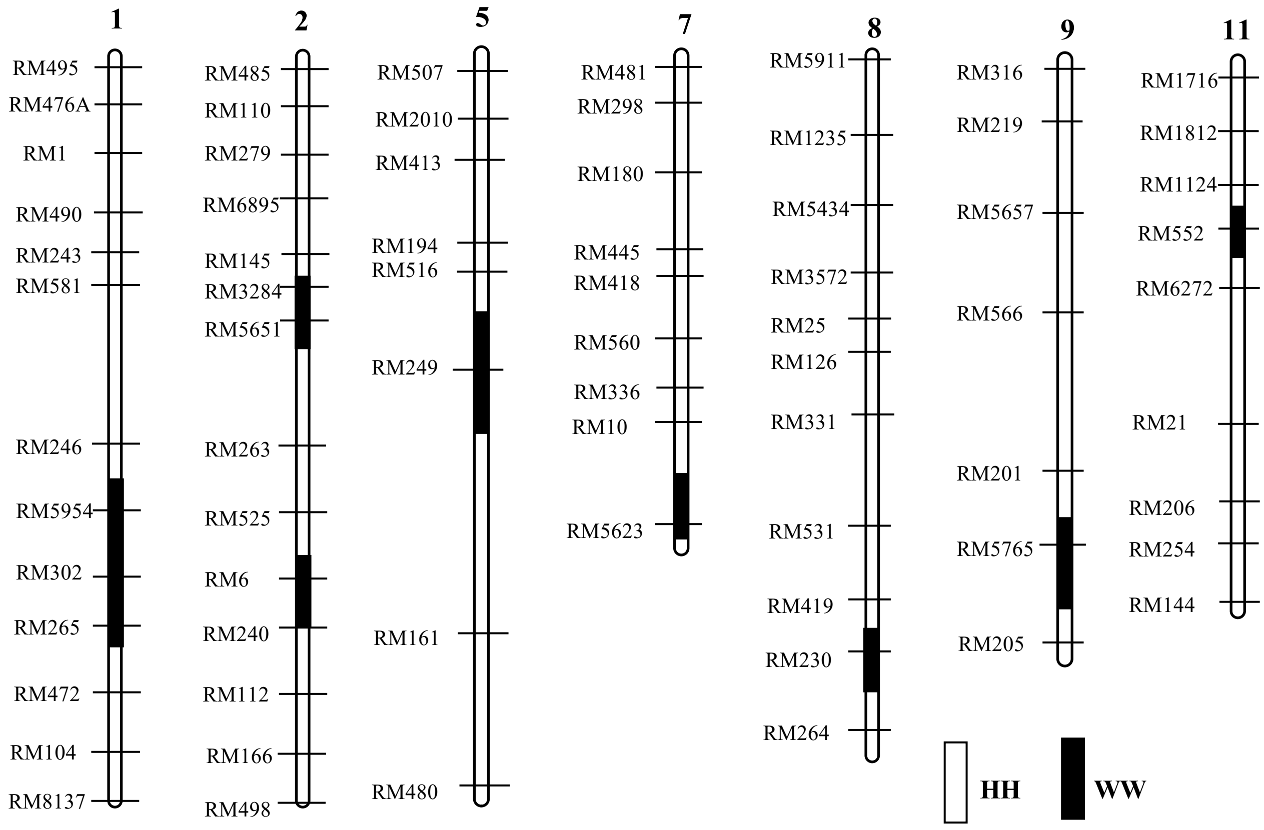

Supplementary Figure 2. Graphical genotype of an introgression line, CR6078. HH, and WW indicate Hwayeong and W1944 homozygousregions, respectively.

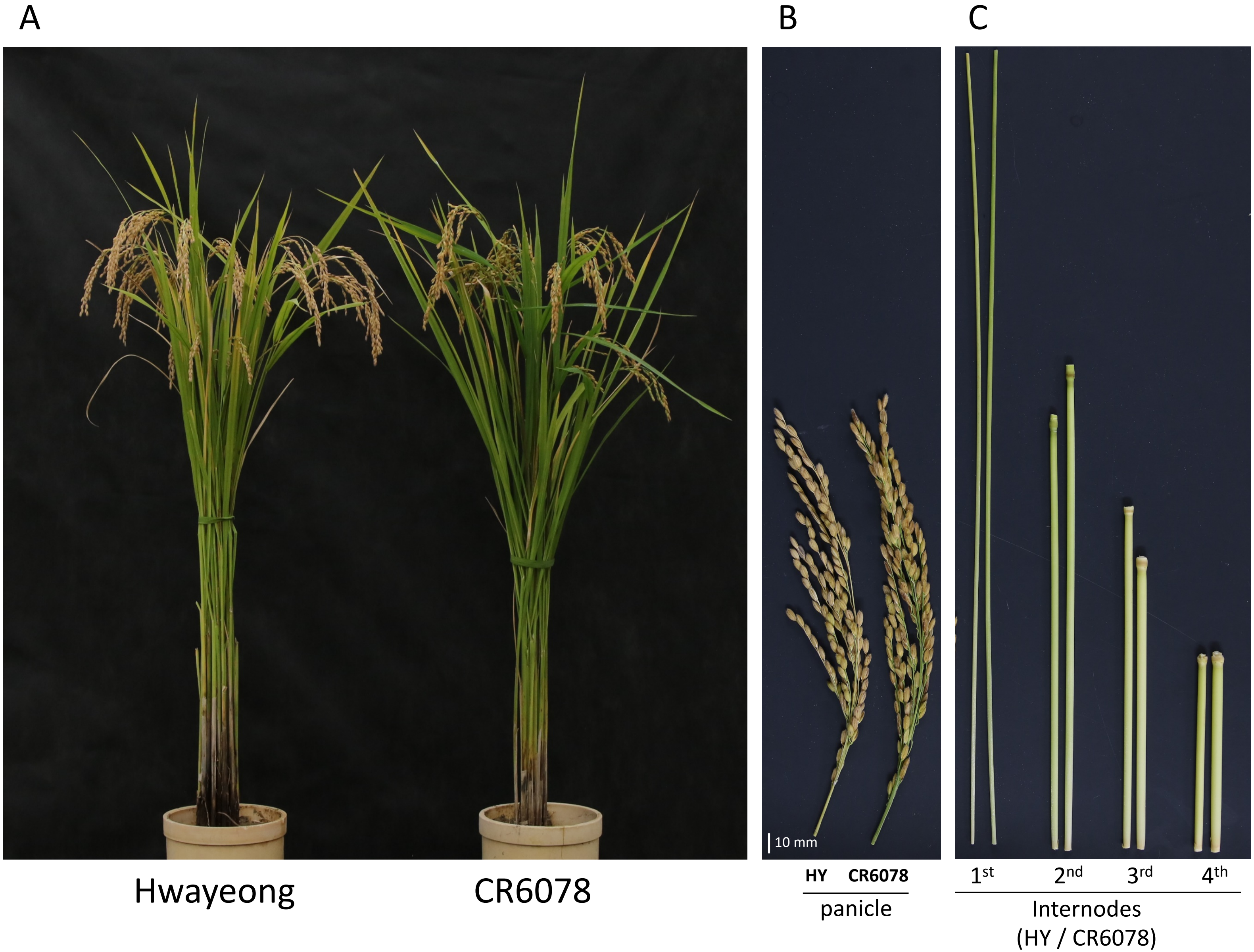

Supplementary Figure 3. Comparison of Hwayeong (HY) and CR6078 for rice plant (A), panicle (B) and internodes (C).

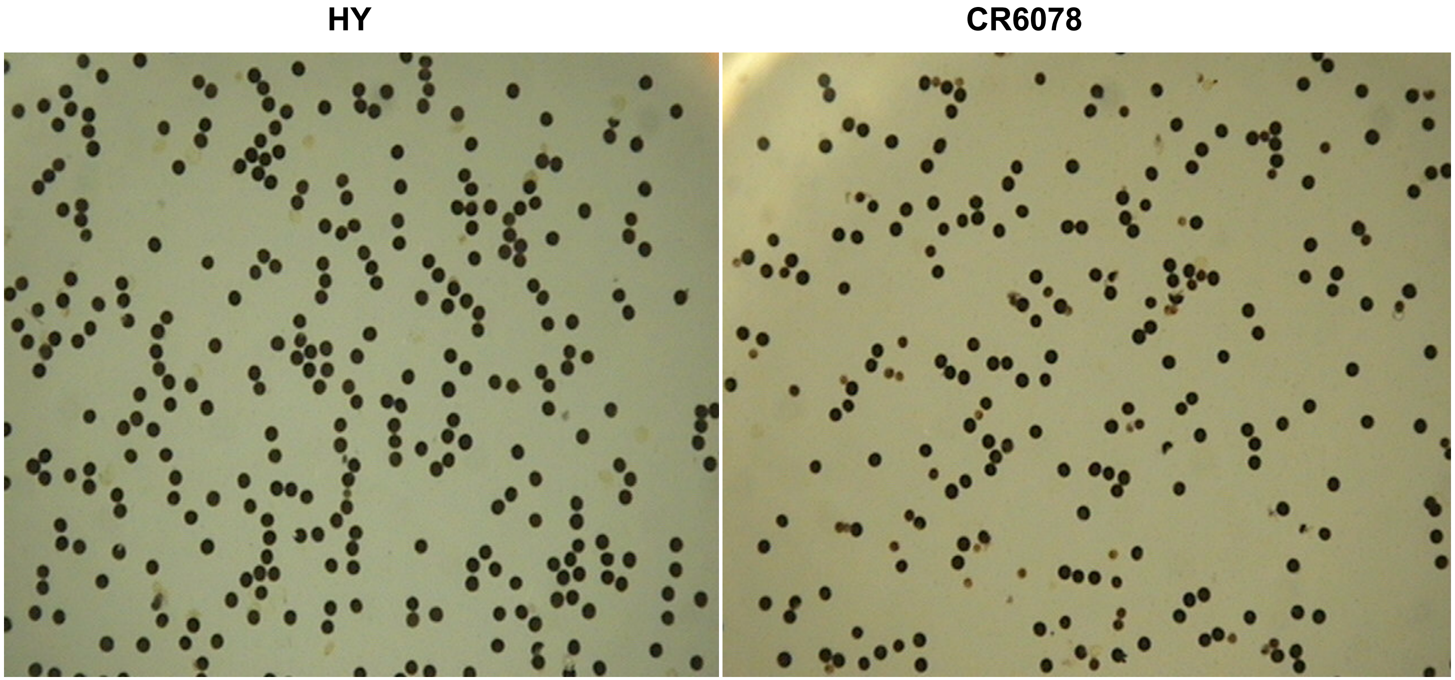

Supplementary Figure 4. Pollen grains were stained with 1% KI-I2 solution and checked w ith microscope. Pollen of HY anCR6078 were normal in morphology and viability.

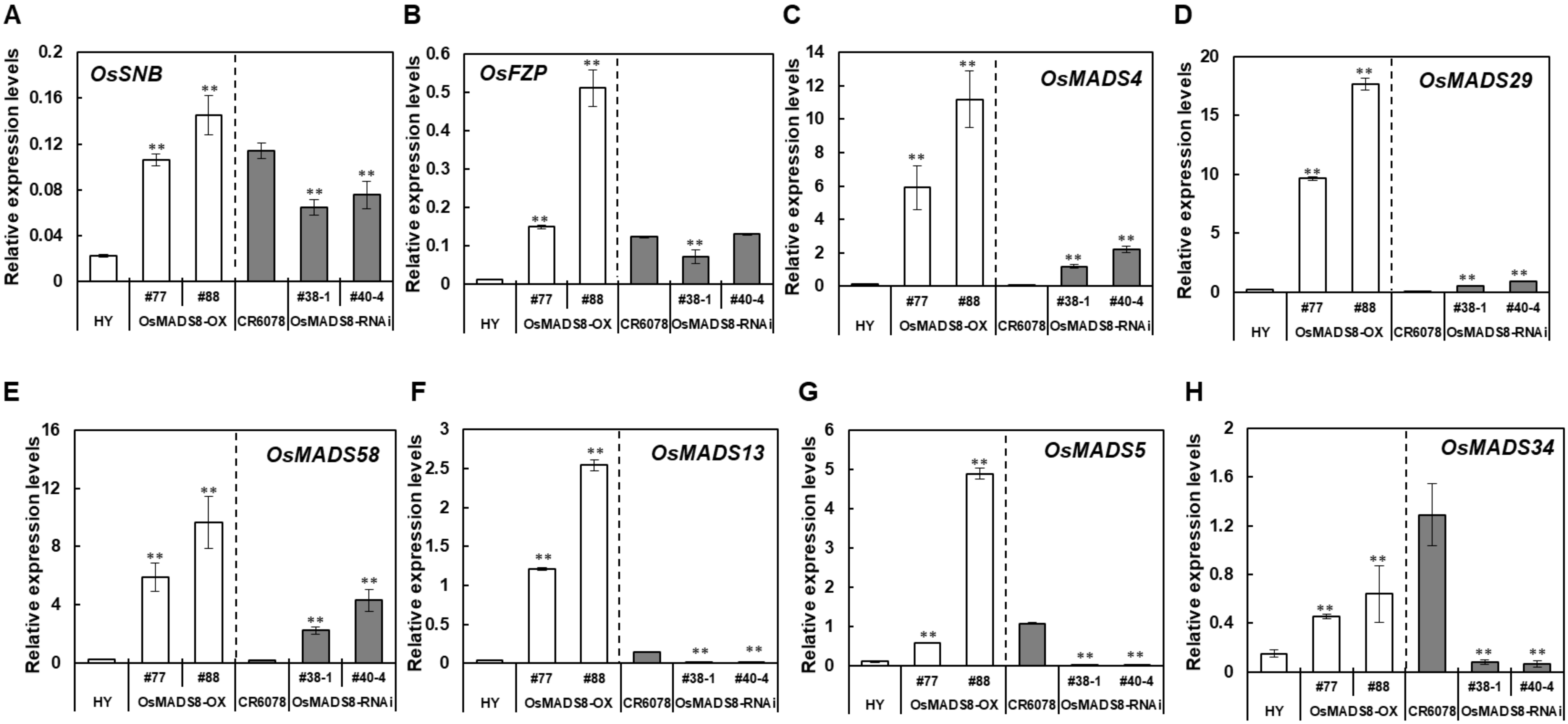

Supplementary Figure 5. Expression of floral organ determination genes in *OsMADS8* transgenic lines. (A, B) A-class, (C, D) B-class, (E) C-class. (F) D-class, (G-H) E-class genes. Total RNA was isolated from 7-day-old seedlings of *OsMADS8* transgenic lines. The error bars represent means ±SD (n=3). Significant differences between HY and each OX line and between CR6078 plants and each RNAi line at *p<0.05 and **p<0.01, respectively

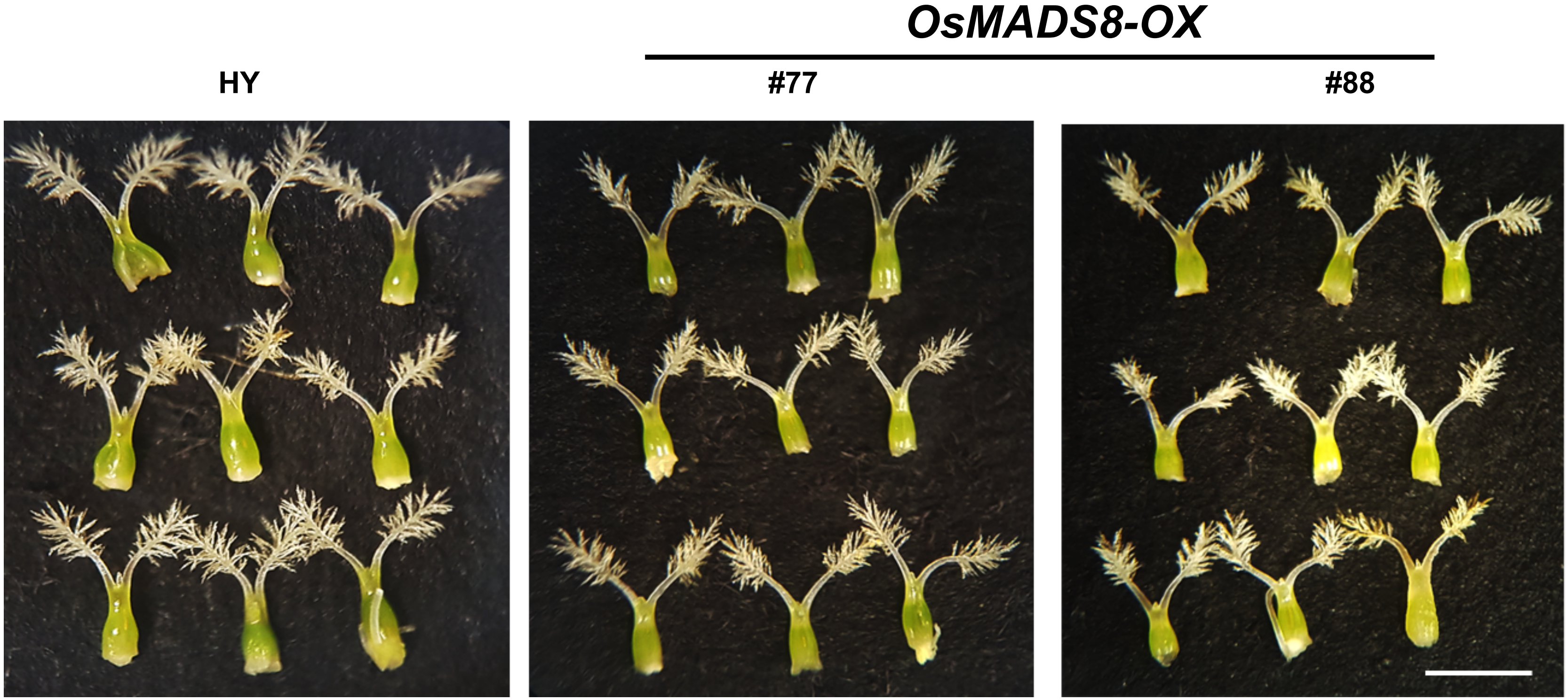

Supplementary Figure 6. Characterization of the pistil phenotype of HY and two *OsMADS8-OX* transgenic lines.

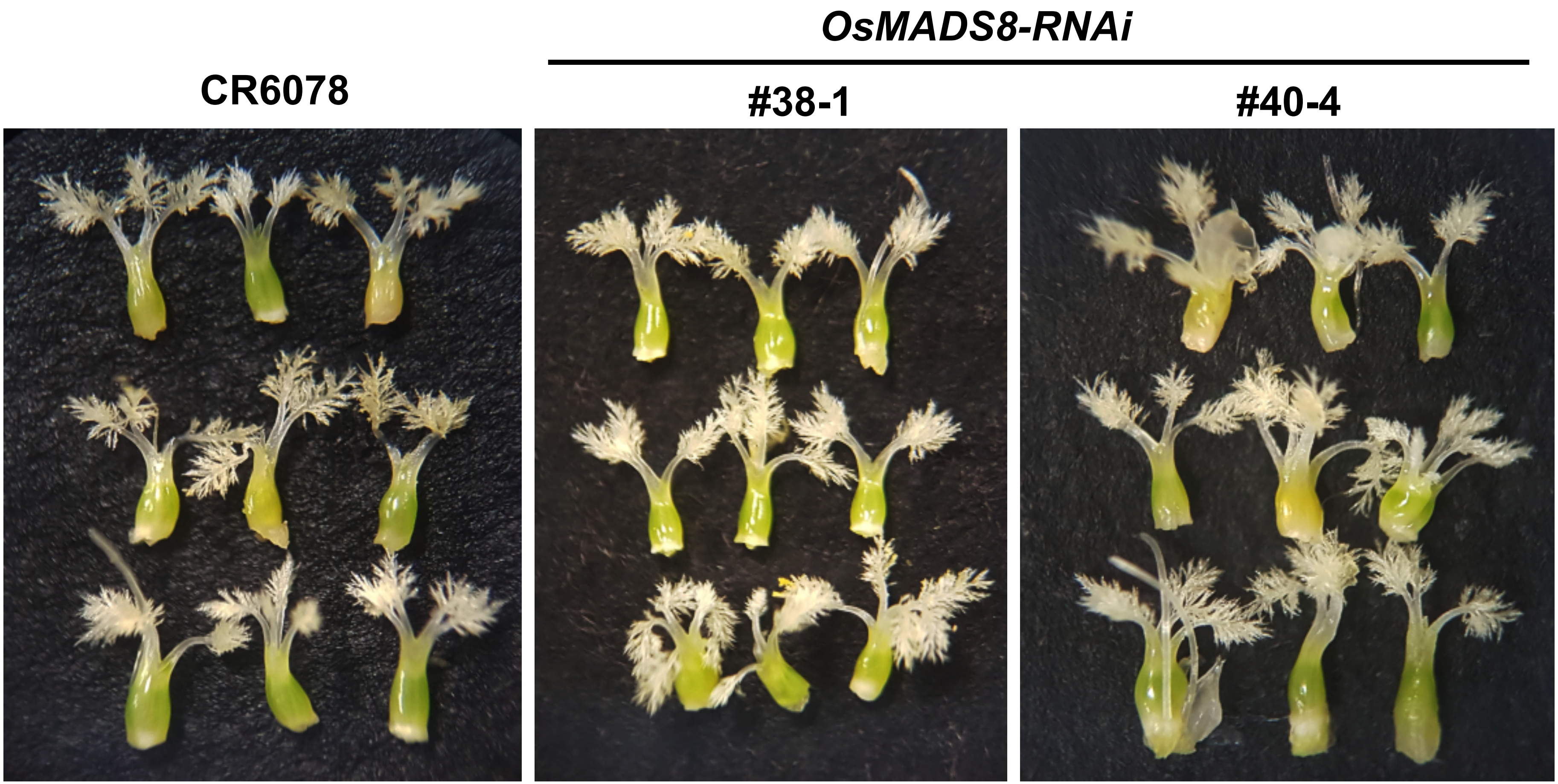

Supplementary Figure 7. Characterization of the pistil phenotype of CR6078 and two *OsMADS8-RNAi* lines.

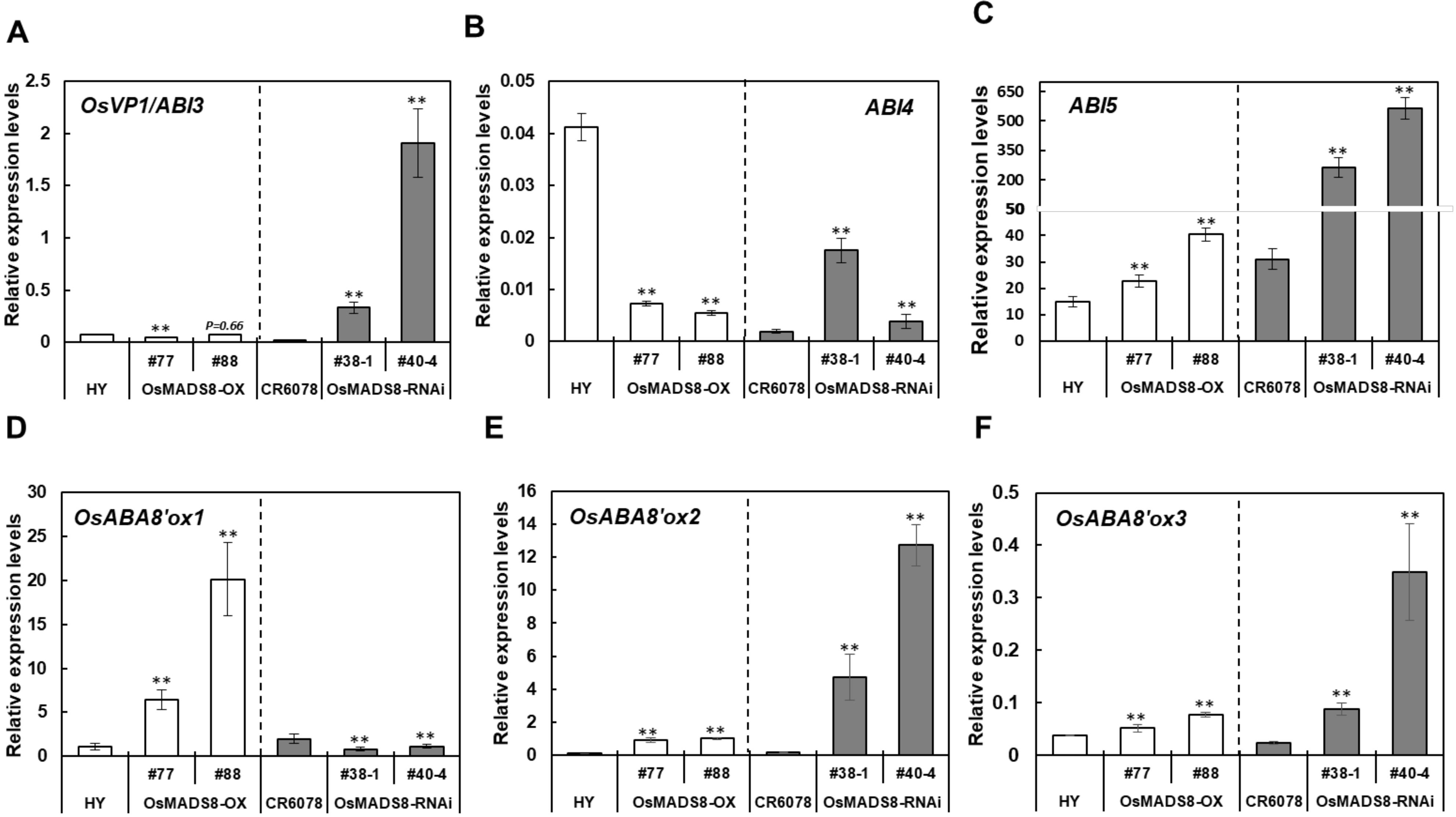

Supplementary Figure 8. Expression of ABA signal transduction and ABA levels catabolism-related genes in *OsMADS8-OX* (#77, #88) and *OsMADS8-RNAi* (#38-1, #40-4) (A-F). Total RNA was isolated from 7-day-old seedlings of *OsMADS8* transgenic lines. Relative expression level of each gene was quantified by real-time PCR and normalized using *OsTMP1* as an internal control. The error bars represent means ±SD (n= 3). Significant differences between HY plants and each OX line, and between CR6078 plants and each RNAi line at *p<0.05 and **p<0.01.
